# Analysis of dairy cattle movements in the northern region of Thailand

**DOI:** 10.3389/fvets.2022.961696

**Published:** 2022-10-04

**Authors:** Sukolrat Boonyayatra, Yuanyuan Wang, Tawatchai Singhla, Apisek Kongsila, Kimberly VanderWaal, Scott J. Wells

**Affiliations:** ^1^Department of Food Animal Clinic, Faculty of Veterinary Medicine, Chiang Mai University, Chiang Mai, Thailand; ^2^Department of Veterinary Population Medicine, College of Veterinary Medicine, University of Minnesota, St. Paul, MN, United States; ^3^The 5th Regional Livestock Office, Department of Livestock Development, Chiang Mai, Thailand

**Keywords:** dairy cattle, movement, network, social network analysis, northern Thailand

## Abstract

Dairy farming in northern Thailand is expanding, with dairy cattle populations increasing up to 8% per year. In addition, disease outbreaks frequently occur in this region, especially foot-and-mouth disease and bovine tuberculosis. Our goal was to quantify the underlying pattern of dairy cattle movements in the context of infectious disease surveillance and control as movements have been identified as risk factors for several infectious diseases. Movements at district levels within the northern region and between the northern and other regions from 2010 to 2017 were recorded by the Department of Livestock Development. Analyzed data included origin, destination, date and purpose of the movement, type of premise of origin and destination, and type and number of moved cattle. Social network analysis was performed to demonstrate patterns of dairy cattle movement within and between regions. The total numbers of movements and moved animals were 3,906 and 180,305, respectively. Decreasing trends in both the number of cattle moved and the number of movements were observed from 2010 to 2016, with increases in 2017. The majority (98%) of the animals moved were male dairy calves, followed by dairy cows (1.7%). The main purpose of the movements was for slaughter (96.3%). Most movements (67.4%) were shipments from central to northern regions, involving 87.1% of cattle moved. By contrast, 56% of the movements for growing and selling purposes occurred within the northern region, commonly involving dairy cows. Constructed movement networks showed heterogeneity of connections among districts. Of 110 districts, 28 were found to be influential to the movement networks, among which 11 districts showed high centrality measures in multiple networks stratified for movement purposes and regions, including eight districts in the northern and one district in each of the central, eastern, and lower northeastern regions of Thailand. These districts were more highly connected than others in the movement network, which may be important for disease transmission, surveillance, and control.

## Introduction

Milk production is projected to be the fastest growing agricultural commodity from 2021 to 2030. In March 2022, the international dairy prices marked a 24% increase compared to the same month last year (1). These persistent upward trends are driven by Asian countries, fulfilling almost one-third of the cow's milk production share globally. Behind these numbers, there are smallholder dairy producers, owning 1–5 animals each, which accounted for nearly 80% of milk production in Asia (2). Over 52% of these producers rely on their dairy business as the sole source of income. Livestock infectious disease is not only a direct threat to their livelihood but also an integral part of social order and stability for many developing countries (3).

A major challenge to disease surveillance and control among dairy production is the lack of documentation on animal movement, defined as the transportation of animals among various locations, such as breeding herds, feeding locations, markets, and slaughterhouses. Epidemiological examinations of animal movement data are typically carried out with social network analysis (SNA), a process of investigating interactions among members of a population through the graph theory (4). Combined with incidence data and sequencing data, foot-and-mouth disease (FMD) virus, bovine tuberculosis (bTB), and Johne's disease, among others, quantifications of cattle movement networks can (1) explain between-herd disease transmissions during outbreaks; (2) investigate multi-species transmissions in wildlife reservoirs; and (3) identify hot spots with an increased risk of infection for a cost-effective targeted surveillance (5–7).

Dairy farming in northern Thailand is expanding, with dairy cattle populations increasing up to 8% per year (8). In addition, disease outbreaks frequently occur in this region, especially FMD (9) and bTB (10, 11). Previously, we collected bTB case data from dairy-intensive areas such as Chiang Mai and Chiang Rai provinces of northern Thailand and found that farms importing cows from dealers in central Thailand had two times higher risk of infection than farms that purchased from other regions (11). Moreover, purchases made through dealers were associated with four times the higher risk of infection than purchases made directly between farms. These factors indicate the importance of connections between components of a network, such as farmers and dealers, in disease transmission, and the need to characterize these patterns to facilitate the development of control programs.

The objective of this study was to describe patterns of dairy cattle movements in the northern region of Thailand. This descriptive analysis can provide analytical context to identify key areas or districts with a high potential for disease transmission among dairy cattle in the northern region of Thailand and the surrounding regions where movement data are not available.

## Materials and methods

### Data of dairy cattle movement

**Figure 1:**
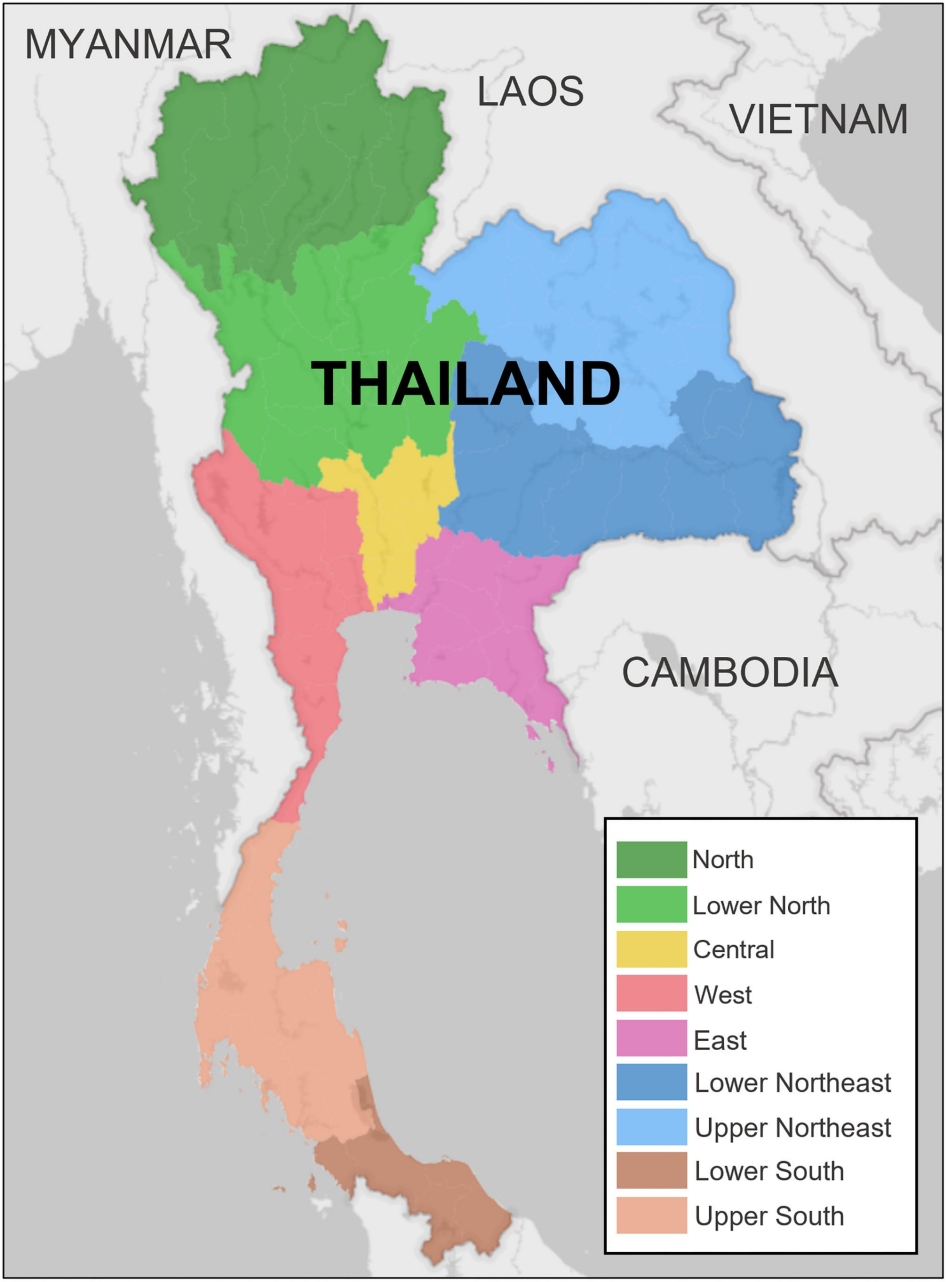
Map of Thailand illustrating the geographical distribution of the study area. Data used for the analysis involved the registered movement of dairy cattle across 110 districts from 32 of 77 provinces in seven of the nine regions of Thailand, including north, lower north, central, west, east, lower northeast, and upper northeast.

All data involving dairy cattle that moved between districts within eight provinces in the northern regions of Thailand from 2010 to 2017 were obtained through collaboration with the Department of Livestock Development (DLD) in Thailand. The collected information included the date of movement, origin and destination locations (at district level), number and type of cattle moved, type of destination, and primary purpose of movement. In Thailand, the geographic hierarchy, ranked by specificity, includes regions, provinces, districts, and farms within districts. Specifically, the cattle movement dataset contains all legal movements that crossed a district border during the study period, including (1) from outside of the northern region of Thailand (including the lower north, central, east, lower northeast, upper northeast, and west regions of Thailand) to the northern region (Figure 1); and (2) within the northern region of Thailand (provinces of Chiang Rai (CR), Chiang Mai (CM), Lamphun (LP), Lampang (LPA), Mae Hong Son (MS), Nan (NA), Phrae (PR), and Phayao (PY)). Cattle movements within districts were not available. A complete list of districts, provinces, and regions is provided in Supplementary Table 2.

### Data analysis

The analysis was carried out at two levels: regions and districts. The number of movements was summarized by year, the type of dairy cattle transported, the purpose of movement, and the premise type of the origin and destination. In addition, an identical analysis was performed on the subset of data that were non-abattoir movements as these movements might be associated with a greater risk of disease transmission between farms. Types of transported cattle included (1) bull, (2) dairy calf with no recorded sex, (3) female dairy calf, (4) male dairy calf, and (5) dairy cows. Primary purposes of the movement included (1) slaughtering, (2) growing, (3) selling, (4) export, and (5) semen collection. Types of premises of the origin and destination included (1) abattoir, (2) house, (3) farm, and (4) others, including market, government office, academic institute, dairy cooperative, private company, quarantine office, and temple. In many cases in Thailand, “farm” where the animals are kept and “house” where the farm owners live are at the same address. Because of the ambiguity of these two terms, data referring to these two terms were pooled together for analysis.

Social network analysis was performed to characterize the movements of dairy cattle in northern Thailand. The analysis was stratified according to the primary purpose of the movement. Identifying influential districts associated with different purposes (i.e., growing, selling, or slaughtering) is essential for disease control surveillance because when an outbreak was detected in a slaughterhouse or a market, a purpose-specific network can be used for contact tracing to identify the source of infection as well as other locations exposed to transmission. In addition, not all documentation is perfect, especially for smallholder dairy producers with backyard farms. For example, documentation is better for abattoir movements to slaughterhouses than growing or selling movements to nearby farms. In addition, a previous study revealing that purchasing dairy cattle from the central region was a significant factor for bTB in dairy cattle farms in northern Thailand (11), indicating the importance of dairy cattle movements from different geographical regions for the disease outbreak. Therefore, the purpose-specific and region-specific networks can reveal detailed connections, which may be otherwise masked by the full network.

Given these justifications, a total of six north-centric weighted and directed networks were constructed: (1) a full network; (2) two purpose-specific networks including (a) growing and selling and (b) slaughtering; and (3) three region-specific networks including (a) movement from the central region to the northern region, (b) movement within the northern region, and (c) movement from other regions to the northern region. In each network, the nodes were districts, and the edges with directions were animal movements between the originating districts and the destination districts. The weights referred to the number of animals transported. The network analyses were performed using R version 4.2.0 with the package “igraph” version 1.3.1. A reproducible notebook including a full list of packages used are accessible at https://github.com/yyw-informatics/thailand_movement_network_analysis.

To capture the overall network structure, global metrics including density, mean path length, and transitivity were calculated. *Density* was measured by the ratio between observed movements and total possible movements among districts. Networks with high density are expected to have increased susceptibility in disease transmission. Networks with shorter *mean path length* (where the pathlength is the number of districts that must be passed through to connect any two districts in the network) could be an indication of a faster spread of infection. In addition, to identify interconnected clusters of districts, the network *transitivity*, defined as the ratio between the observed number of closed triplets and the total possible number of closed triplets, was calculated.

Network metrics were calculated to identify districts either with high movement frequency or positioned at important locations within the network that could disproportionately influence the spread between other districts, for example, bridging separated districts. Such districts were considered influential hot spots as they were expected to have a higher probability of becoming infected and transmitting diseases than other districts in the network. For each network, local metrics, including degree centrality, eigen centrality, strength, betweenness, closeness, and reciprocity were calculated to describe district-level connectivity and identify influential districts with an increased risk of infection and transmission.

- *Degree centrality* measured the number of direct connections held by each district, with in-degree referring to the number of inbound movements and out-degree referring to the number of outbound movements. *Eigen centrality* extended the degree centrality by calculating the second-order connections of districts. Districts with a high degree or high Eigen centrality indicated an increased frequency of movement, which may indicate an increased risk of disease exposure and onward transmission to other districts.
- As the number of animals associated with each movement was recorded in the data, *strength* was measured by summing up the weights associated with each edge. Districts with high strength might not have a high degree, but they could be associated with an increased probability of spreading infectious diseases due to the large volume of animals transported.
- *Betweenness centrality* measures the number of edges that traverse a district through the shortest path between each pair of districts. It measures the bridging effect of each district in the network. Districts with high betweenness might not have high frequencies of animal movement, but they acted like links between otherwise disconnected districts.
- *Closeness centrality* is the mean of distances of the shortest path between each pair of districts. It measures the extent to which a district is in the central position of a movement network. Districts with high closeness were expected to be well-connected with other districts in the network; hence, these districts have the potential to facilitate “super-spreading” transmissions in a star-like network.
- *Reciprocity* measures the likelihood of districts to be mutually connected. During outbreak investigations, districts with reciprocal movements suggested that both districts can be the source and destination, thus indicating an increased risk of infection and transmission.

Finally, districts were ranked based on the metrics discussed previously for each of the six networks. Using each of the centrality metrics, influential districts were selected if their centrality measures had exceeded the mean plus two standard deviations for that metric in the corresponding network. These districts either had high movement frequency or were essentially located, which can influence the flow of movement among separated clusters of districts. These districts were expected to be associated with an increased risk of infection and disease transmission. The results of these selected districts were first visualized in a heatmap showing the following information: (1) For *which network metric(s)*, the district had significant values? This information allowed evaluation of the role of the district in the corresponding network. For example, if the betweenness was high, then the district was bridging multiple separated districts. If the degree was high, then the district had a high frequency of movement. (2) In *how many network(s)* (of the six evaluated) the metric was found to be significant? For example, if a district with a high degree was found in multiple networks, then the selected district was likely to be important. (3) In *which network(s)* (of the six evaluated) the district was found to have significant metrics? This information allowed the discovery of districts that were influential to certain stratified networks, such as the growing and selling network where the movement frequency was much less than the slaughtering network but covered much more districts, which would increase the probability of disease infection and transmission. Geographical maps showing these influential districts and the movement in their corresponding networks were used to demonstrate the spatial distance between these districts. Maps were created using the R package “ggmap” version 3.0.0 through queries of Google Maps.

## Results

### Descriptive summary

#### Movement by year

Data of dairy cattle movements in 110 districts of 32 provinces across seven regions in Thailand were included for the analysis (Figure 1). In total, 3,906 movements were documented, and 180,305 animals were moved (Table 1). Both numbers decreased consistently from 2010 to 2016, starting from 42,124 animals with 832 movements in 2010 to 8,852 with 300 movements in 2016. The only exception was in 2017, when the movements nearly doubled, although the number of animals moved was consistent with the previous 2 years. No apparent seasonality was observed across 12 months with the lowest numbers reported in June (Table 2).

**Table 1:** Overall trend of the number of dairy cattle moved (animal head) and the number of dairy cattle movements (frequency) between districts in northern Thailand from 2010 to 2017.

**Number**	**2010**	**2011**	**2012**	**2013**	**2014**	**2015**	**2016**	**2017**	**Total**
Cattle moved	42,124 (23.4%)	34,589 (19.2%)	35,455 (19.7%)	23,796 (13.2%)	14,802 (8.2%)	10,454 (5.8%)	8,852 (4.9%)	10,233 (5.7%)	180,305 (100%)
Movements	832 (21.3%)	562 (14.4%)	554 (14.2%)	428 (11.0%)	339 (8.7%)	310 (7.9%)	300 (7.7%)	581 (14.9%)	3,906 (100%)

**Table 2:** Distribution of dairy cattle moved (animal head) and movements (frequency) between districts in northern Thailand from 2010 to 2017 stratified by month.

**Month**	**Movements**	**Cattle moved**
January	327 (8.37%)	15,355 (8.52%)
February	294 (7.53%)	14,239 (7.90%)
March	349 (8.93%)	15,854 (8.79%)
April	354 (9.06%)	17,712 (9.82%)
May	291 (7.45%)	14,300 (7.93%)
June	251 (6.43%)	10,419 (5.78%)
July	295 (7.55%)	12,709 (7.05%)
August	307 (7.86%)	14,212 (7.88%)
September	341 (8.73%)	16,784 (9.31%)
October	361 (9.24%)	17,263 (9.57%)
November	331 (8.47%)	15,319 (8.50%)
December	405 (10.37%)	16,139 (8.95%)
Total	3,906 (100.00%)	180,305 (100.00%)

#### Movement by cattle type

The animals moved were predominantly male dairy calves (98.0%, Table 3). The number of male dairy calf movements decreased during this time, from over 41,000 in 2010 to about 9,000 in 2017. The second most common cattle type moved was dairy cows (3,009 animals). The overall trend was decreasing for most animal types, except for bulls and dairy cows, both of which decreased from 2010 to 2016 and increased from 2016 to 2017.

**Table 3:** Distribution of the different cattle types moved (animal head) between districts in northern Thailand from 2010 to 2017.

	**2010**	**2011**	**2012**	**2013**	**2014**	**2015**	**2016**	**2017**	**Total**
Bull	21 (0%)	1 (0%)	1 (0%)	12 (0%)	28 (0.2%)	9 (0.1%)	9 (0.1%)	187 (1.8%)	268 (0.1%)
Dairy calf ^a^	80 (0.2%)	32 (0.1%)	0 (0%)	0 (0%)	0 (0%)	0 (0%)	0 (0%)	0 (0%)	112 (0.1%)
Female dairy calf	41 (0.1%)	27 (0.1%)	19 (0%)	55 (0.2%)	17 (0.1%)	39 (0.4%)	13 (0.1%)	8 (0.1%)	219 (0.1%)
Male dairy calf	41,071 (97.3%)	34,018 (98.5%)	35,128 (99.2%)	23,578 (99.1%)	14,471 (97.8%)	10,272 (98.2%)	8,792 (99.3%)	9,330 (91.2%)	176,660 (98.0%)
Dairy cow	991 (2.3%)	463 (1.3%)	275 (0.8%)	151 (0.6%)	286 (1.9%)	134 (1.3%)	38 (0.4%)	708 (6.9%)	3,046 (1.7%)
Total	42,204 (100%)	34,541 (100%)	35,423 (100%)	23,796 (100%)	14,802 (100%)	10,454 (100%)	8,852 (100%)	10,233 (100%)	180,305 (100%)

a Number of dairy calves with no specific gender recorded.

#### Movement by purpose

The main primary purpose of the movement was slaughter, which accounted for 82.0% of all movements, contributing 96.3% of all cattle moved, followed by growing (12.2% of movements) and selling (5.7% of movements), as shown in Table 4. When evaluated by year (Table 4), slaughter accounted for at least 78% of cattle movements each year until 2016. In 2017, there was a different pattern of growing, selling, and slaughter, with 22, 24, and 53%, respectively.

**Table 4:** Number of dairy cattle moved (animal head) and movements (frequency) between districts in northern Thailand for different primary purposes from 2010 to 2017.

**Purpose**	**Cattle (%)**	**Movements (%)**	**2010**	**2011**	**2012**	**2013**	**2014**	**2015**	**2016**	**2017**
Slaughtering	173,552 (96.3%)	3,195 (81.8%)	687 (82.6%)	497 (88.4%)	511 (92.2%)	389 (90.9%)	307 (90.6%)	263 (84.8%)	233 (77.7%)	308 (53%)
Growing	5,229 (2.9%)	484 (12.4%)	101 (12.1%)	56 (10%)	36 (6.5%)	36 (8.4%)	28 (8.3%)	45 (14.5%)	51 (17%)	131 (22.5%)
Selling	1,500 (0.8%)	225 (5.8%)	44 (5.3%)	9 (1.6%)	7 (1.3%)	3 (0.7%)	4 (1.2%)	2 (0.6%)	16 (5.3%)	140 (24.1%)
Export	20 (0%)	1 (0%)	0%	0%	0%	0%	0%	0%	0%	1 (0.2%)
Semen	4 (0%)	1 (0%)	0%	0%	0%	0%	0%	0%	0%	1 (0.2%)
collection
Total	180,305	3,906	832	562	554	428	339	310	300	581

#### Movement by destination

Abattoir was the most common destination, accounting for 77.4% of cattle moved (Table 5). The second most common destination was house or farm, accounting for 30.4% of movements and 22.4% of cattle moved. Across 8 years of study, the most frequent destination was the abattoir, except for 2010, followed by house or farm (Table 5). A comparison between the destination and the purpose of movements (Table 4) revealed that a total of 34,673 animals were moved for slaughtering purposes to non-abattoir premises.

**Table 5:** Number of dairy cattle movements (frequency) and dairy cattle moved (animal head) between districts in northern Thailand to different premises of destination from 2010 to 2017.

**Premises of destination**	**Number**	**2010**	**2011**	**2012**	**2013**	**2014**	**2015**	**2016**	**2017**	**Total**
Abattoir	Cattle	5,843 (13.9%)	34,008 (98.3%)	34,967 (98.6%)	22,989 (96.6%)	14,475 (97.8%)	9,922 (94.9%)	8,143 (92.0%)	9,116 (89.1%)	139,463 (77.3%)
	Movements	138 (16.6%)	498 (88.6%)	516 (93.1%)	389 (90.9%)	307 (90.6%)	263 (84.8%)	233 (77.7%)	306 (52.7%)	2,650 (67.8%)
Farm/House	Cattle No.	36,090 (85.7%)	550 (1.6%)	485 (1.4%)	787 (3.3%)	252 (1.7%)	460 (4.4%)	703 (7.9%)	1,078 (10.5%)	40,405 (22.4%)
	Movements	663 (79.7%)	60 (10.7%)	37 (6.7%)	36 (8.4%)	26 (7.7%)	40 (12.9%)	64 (21.3%)	265 (45.6%)	1,191 (30.5%)
Others	Cattle No.	191 (0.4%)	31 (0.1%)	3 (0%)	20 (0.1%)	75 (0.5%)	72 (0.7%)	6 (0.1%)	39 (0.4%)	437 (0.2%)
	Movements	31 (3.7%)	4 (0.7%)	1 (0.2%)	3 (0.7%)	6 (1.8%)	7 (2.3%)	3 (1.0%)	10 (1.7%)	65 (1.7%)
Total	Cattle No.	42,124	34,589	35,455	23,796	14,802	10,454	8,852	10,233	180,305
	Movements	832	562	554	428	339	310	300	581	3,906

Considering the movements to abattoirs as terminal movements of live animals, which are of less importance for some disease transmission, the movements to abattoirs were filtered out and reanalyzed to reveal the pattern of dairy cattle movements to other types of destinations. Among non-abattoir premises as destinations, house or farm was the predominant destination of movements, contributing 80.6 to 97.4% of these movements. House or farm also contributed over 76% of cattle moved to locations other than abattoirs (Table 6). In many of these non-abattoir movements, dairy cows were the most common cattle type moved (depending on the year, 12.3 to 78.9% of movements and 2.1 to 90.8% of cattle moved). The numbers of farms and houses associated with the dairy cattle movement in each district are provided in Supplementary Tables 1, 3, respectively.

**Table 6:** Number of dairy cows moved (animal head) among the total cattle moved (animal head) and their corresponding movements (frequency) by types of premises of destination other than abattoirs in northern Thailand from 2010 to 2017.

**Premises of**
**destination**	**Number of animals and movements**	**2010**	**2011**	**2012**	**2013**	**2014**	**2015**	**2016**	**2017**	**Total**
Farm/	Cattle	36,090	550	485	787	252	460	703	1,078	40,405
House	Dairy cow	755	429	219	132	227	68	29	505	2,364
	% of dairy cow	2.1	78.0	45.2	16.8	90.1	14.8	4.1	46.8	5.9
	Movements	663	60	37	36	26	40	64	265	1,191
	Dairy cow movements	100	47	26	17	18	14	8	161	391
	% of dairy cow movements	15.1	78.3	70.3	47.2	69.2	35.0	12.5	60.8	32.8
Others	Cattle	191	31	3	20	75	72	6	39	437
	Dairy cow	158	30	3	10	55	63	0	25	344
	% of dairy cow	82.7	96.8	100.0	50.0	73.3	87.5	0	64.1	78.7
	Movements	31	4	1	3	6	7	3	10	65
	Dairy cow movements	23	3	1	2	4	4	0	6	43
	% of dairy cow movements	74.2	75.0	100.0	66.7	66.7	57.1	0	60.0	66.2

#### Movement frequency across regions

In northern Thailand, the majority of the movements (67.3%) was from the central region, accounting for 87.1% of cattle moved into northern Thailand from elsewhere (Table 7). Data showed that animals involved in these movements were predominantly male dairy calves for slaughtering, which totaled 155,802 animals and accounted for 99.2% of all animals transported from central to northern Thailand.

**Table 7:** Dairy cattle movements (frequency) and the number of cattle moved (animal head) between districts in northern Thailand with different regions as origin and destination from 2010 to 2017.

**Regions**	**Origin**		**Destination**	
	**Movements**	**Cattle moved**	**Movements**	**Cattle moved**
Central	2,629 (67.3%)	157,054 (87.1%)	2 (0%)	20 (0%)
Upper North	563 (14.4%)	1,652 (0.9%)	3,891 (99.6%)	180,135 (99.9%)
Lower North	31 (0.8%)	129 (0.1%)	6 (0.2%)	37 (0%)
Upper Northeast	0 (0%)	0 (0%)	1 (0%)	2 (0%)
Lower Northeast	440 (11.3%)	14,517 (8.1%)	2 (0%)	7 (0%)
East	196 (5.0%)	6,368 (3.5%)	0 (0%)	0 (0%)
West	47 (1.2%)	585 (0.3%)	4 (0.1%)	104 (0.1%)
Total	3,906 (100%)	180,305 (100%)	3,906 (100%)	180,305 (100%)

#### Movement within northern Thailand

In 2017, the number of movements within the northern region was nearly 10 times higher than that the previous year, and it has exceeded the total from the central region for the first time since 2010 (Supplementary Figure 1). Over half of the movements were the movements of dairy cows (56%) for growing and selling purposes (69%) to house, which accounted for 63% of the documented destinations.

### Network analysis on a district level

#### The heterogeneous distribution of network connectivity among districts

Overall, most districts were active as nearly 70% had more than one movement. However, the level of connectivity was different. Graph-level network metrics over the study years, from 2010 to 2017, are summarized in Table 8. No significant temporal signal was observed. On the district level, the distributions of most local network metrics were highly skewed (Supplementary Figure 2), suggesting the presence of several highly connected districts. For example, in the full network, the mean degree was 70.38, with a median of 4, due to four districts having more than 500 movements. Similar distributions were observed for other metrics including eigen centrality, betweenness, closeness, and strength.

**Table 8:** Graph-level network metrics for full network and networks stratified for year, region, and primary purpose of dairy cattle movements between districts in northern Thailand from 2010 to 2017.

**Networks**		**In degree**	**Out degree**	**Degree**	**Eigen centrality**	**Betweenness**	**Closeness**	**Reciprocity**	**Strength**	**Graph density**	**Transitivity**	**Average path length**
Overall	2010–2017	21.18	17.85	10.54	0.99	0.1	0.3	0.01	70.38	0.32	0.19	3.07
By year	2010	9.71	9.92	4.79	0.97	0.1	1.3	0.01	43.79	0.59	0.28	2.28
	2011	7.1	4.53	3.5	0.96	0	1.21	0	24.98	0.28	0.21	1.27
	2012	7.69	6.14	3.8	0.98	0	1.09	0	23.08	0.25	0	1.36
	2013	7.23	6.37	3.54	0.97	0.02	1.03	0.01	23.78	0.34	0.21	1.96
	2014	7.27	6.48	3.6	0.99	0.01	0.95	0	19.37	0.28	0	1.4
	2015	7.67	5.73	3.83	0.99	0.02	0.69	0	17.22	0.25	0	2.11
	2016	10.38	8.25	5.14	0.99	0.05	0.97	0	24	0.5	0.15	1.85
	2017	4.21	3.98	2.05	1	0.07	0.21	0.01	22.35	0.22	0.16	2.93
By region	Central to North	41.57	47.23	23.41	0.99	0	1.18	0	125.19	1.53	0	1
	Other to North	7.64	2.74	3.81	0.99	0	1.22	0	18.94	0.12	0	1.52
	Within North	2.05	2.71	1.29	0.97	0.17	0.33	0.08	20.68	0.2	0.21	2.69
By purpose	Grow and sell	0.88	1.36	0.7	0.99	0.1	1.34	0.03	13.13	0.06	0.17	3.2
	Slaughter	53.34	45.85	26.38	0.98	0.02	1.21	0	152.14	1.86	0.12	2.12

#### The contrasting characteristics of networks stratified for movement purposes

Networks stratified for different movement purposes, either slaughtering (SA network) or growing and selling (GS network), showed contrasting patterns. Because of the large volume of animals and movements, the SA network had a much higher level of strength (mean = 152.14) than the GS network (mean = 13.13), as seen in Table 9. The GS network contained over 97% of districts in the full network (108 of 111 nodes), representing higher district coverage than the SA network (42 nodes). As seen in **Figure 4**, when simplifying the movement edges between two districts into a single edge, the SA network had fewer edges than the GS network Consequently, compared with the SA network, the GS network had higher betweenness (betweenness _GS =_ 50.67 vs. betweenness _SA_ 3.69, respectively) and longer average path length (length _GS =_ 3.2 vs. length _SA_ = 2.12, respectively).

**Table 9:** District-level network metrics for the full network and networks stratified for the primary purpose of dairy cattle movement between districts in northern Thailand from 2010 to 2017.

**Networks**	**Degree^a^**	**Eigen centrality^a^**	**Betweenness^a^**	**Closeness^a^**	**Average path length**
Full	70.38 (4, 2368)	0.03 (0, 1)	52.93 (0, 1240.44)	0.33 (0.33, 0.48)	3.07
Growing, selling	13.13 (3, 162)	0.03 (0, 1)	50.67 (0, 1184.02)	0.34 (0.32, 1)	3.2
Slaughtering	152.14 (2, 2264)	0.06 (0, 1)	3.69 (0, 28)	0.42 (0.31, 1)	2.12

a Values of mean (median, maximum) are shown to describe the district-level network metrics.

#### The influential districts identified for each stratified network

**Figure 2:**
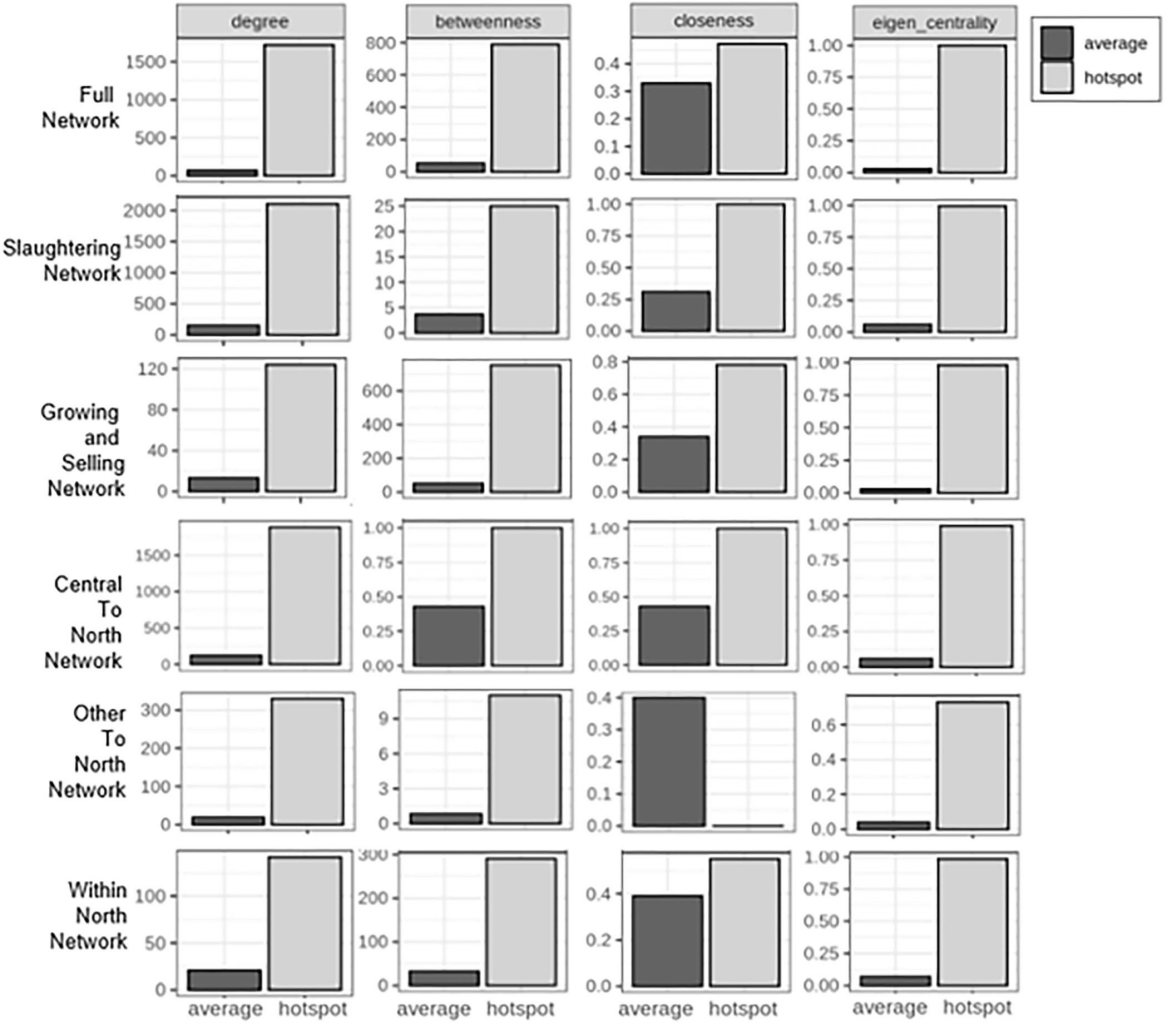
Comparisons of network averages and influential hot spot district averages. This bar plot shows the comparison of means between each of the six networks (full network, slaughtering network, growing and selling network, central-to-north movement, other-to-north movement, and within-north movement). The x-axis represents the groups in comparison: network average or influential hot spot average. The y-axis represents the value of means. This panel contains six rows showing the network evaluated and four columns showing the network metric calculated.

**Figure 3:**
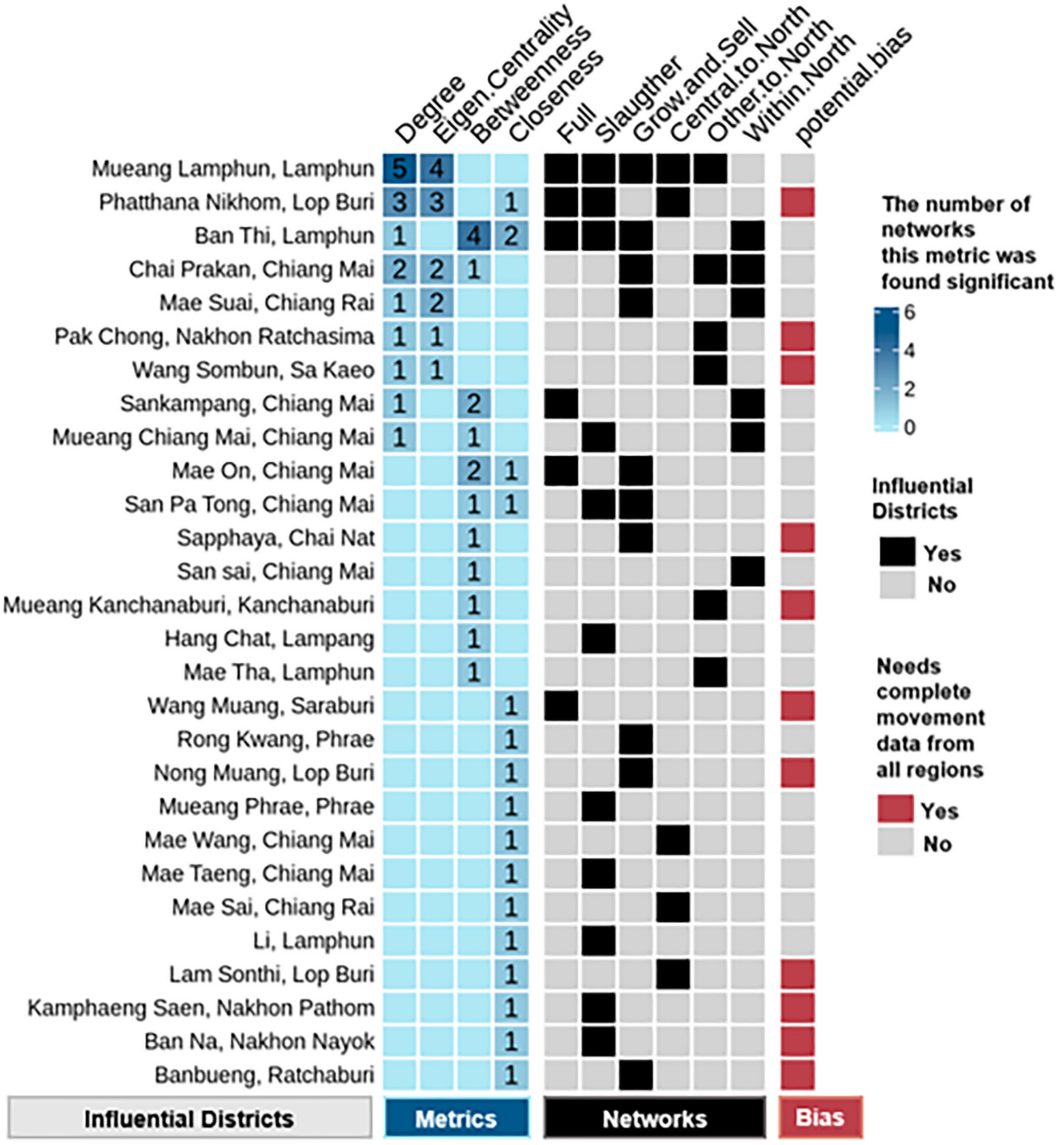
Influential districts are identified through social network analysis. The heatmap has three or four sections: districts, metrics, networks, and bias. The *district* section shows a complete list of districts identified as their network metrics exceeded the network mean plus two standard deviations of the metrics in corresponding networks. The *metric* section shows which of the four metrics were significant and how many networks were associated with these metrics. The *network* section shows which of the six networks the district had significant metrics. The bias section shows if the selected district was from the northern region, or complete movement data are needed to reevaluate its significance.

**Figure 4:**
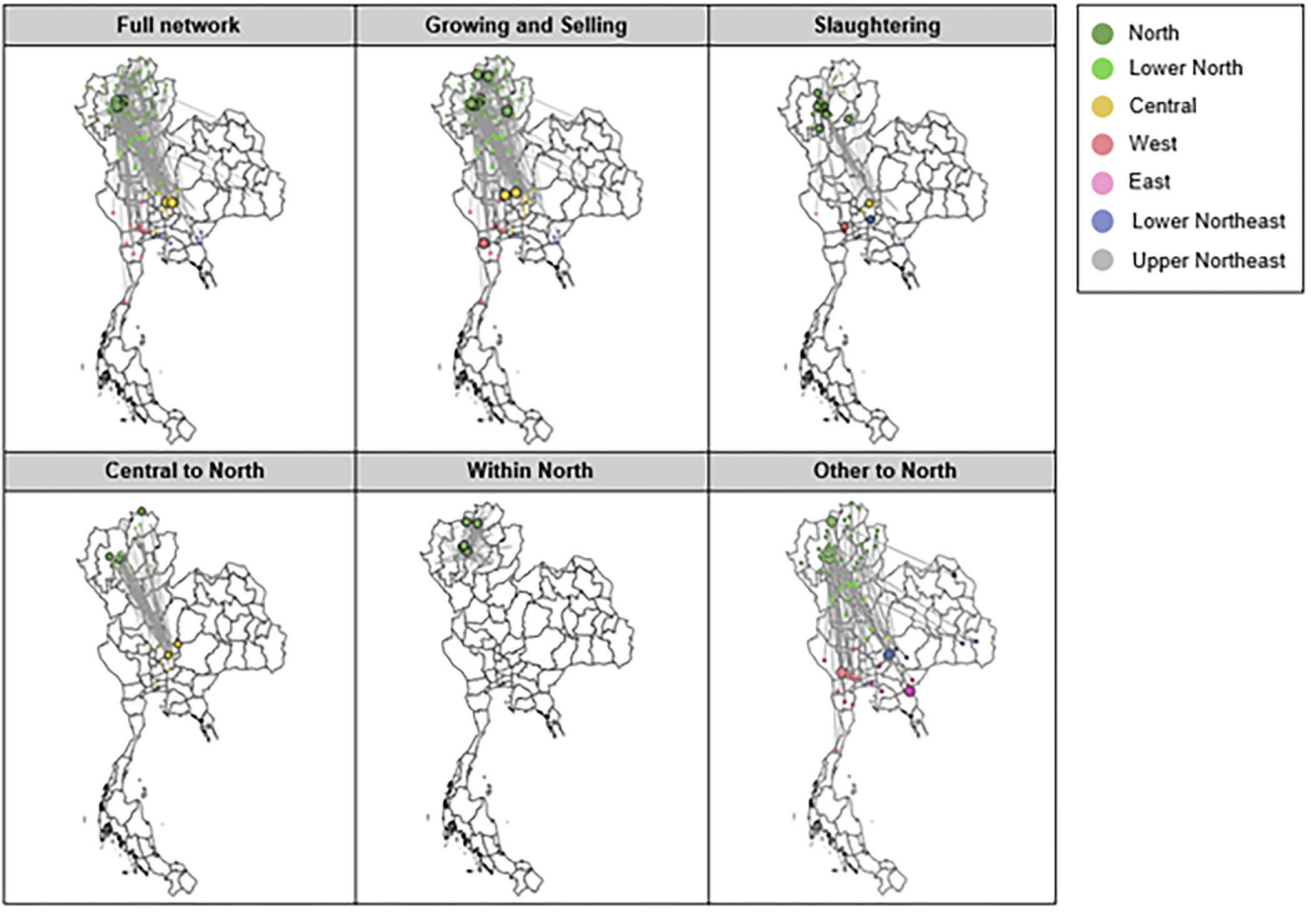
Full network, networks of movements with growing and selling as the primary purposes of movements, networks of movements from central to northern, within northern, and other to northern regions of Thailand, as overlaid on the geographical map of Thailand. Colored dots represent districts in the movement dataset. Big colored circles represent the influential districts of the dairy cattle movement for each network identified through social network analysis. Directed movement edges are simplified to a single edge between each pair of districts for visualization purposes.

Given the two observations mentioned previously, influential districts were identified using the cutoff of network averages plus two standard deviations of each network metrics. This step was repeated for six networks to identify unique districts specific to each network. The comparisons of means between the network averages and the influential hot spot averages for each of the six networks are seen in Figure 2. Of the total 110 districts, 28 were determined to be influential based on their significant values on any of the centrality metrics (degree, eigen centrality, betweenness, and closeness). As seen in Figure 3, a total of 11 districts, including eight districts in the northern and one district in each of the central, eastern, and lower northeastern regions, were found to be significant on multiple metrics in at least one network. For example, Mueang Lamphun district from the Lamphun province in the northern region was found to be important for all five networks, except the within-north network. Specifically, it had a high degree in five networks and high eigen centrality in four networks. In addition, Ban Thi district in Lamphun province in the northern region was important for the full movement network, the slaughtering network, the growing and selling network, and the within-north network. This district showed particularly high betweenness in four networks, high closeness in two networks, and a high degree in one network. Moreover, Chai Prakan district in Chiang Mai province and Mae Suai district in Chiang Rai province were found to have frequent movement in both the growing and selling networks and the within-northern movement network. The geographic locations of these districts are seen in Figure 4 with their corresponding networks.

## Discussion

The results from this study demonstrate the dynamic pattern of cattle movements in northern Thailand. From 2010 to 2016, the movement of dairy cattle together with the number of cattle moved had decreased. This could be influenced by the restriction of cattle movement during this period. The northern region of Thailand is an endemic area of several infectious diseases in cattle, such as FMD and bTB. FMD has been regularly reported in dairy and beef cattle in several provinces in northern Thailand. A previous study on FMD outbreaks from 2015 to 2017 reported an increasing number of FMD outbreaks, which peaked in 2016, while a significant reduction of FMD outbreaks was observed in 2017 (12). In addition, bTB was extensively investigated from 2011 to 2015 in northern Thailand by the DLD (11). The official reports on the detection of these infectious diseases resulted in many restrictions on cattle movement within and across the northern region of Thailand.

Overall, most cattle moved between districts for the purpose of slaughter, primarily male dairy calves. Male dairy calves are considered surplus animals in dairy cattle farming because they do not contribute to milk production. The central region is the most extensive dairy farming area in Thailand (13). Therefore, the exportation of male dairy calves from the central region to other regions of the country is expected. Moreover, male dairy calf is the main ingredient for roasted calf, which is a very popular dish in northern Thailand. Our results revealed that the consumption of male dairy calves in the region is very high, as indicated by the main cattle type imported from other regions of the country. The movements of male dairy calves would be expected to have a low risk of pathogen transmission since animals were shipped to abattoirs for slaughter. Although a significant proportion of these calves intended for slaughter were not shipped directly to abattoirs, many are moved to houses or farms. Information was not available to indicate how long these animals remained at the house or farm location prior to slaughter or whether they were in contact with other cattle in situations that could result in disease transmission. Without movements of male dairy calves to abattoirs, movements of dairy cows to houses or farms for the purpose of growing and selling were dominant. Most of these movements were within northern Thailand, which could be considered a risk for disease transmission in the region.

In 2010, most movements were for the purpose of slaughtering male dairy calves, but they were recorded as farm or house being the destination. This could be explained by two reasons: First, slaughtering male dairy calves was usually conducted at houses or farms at that particular period. The carcasses might be locally sold and consumed in the area close to where the slaughtering process was performed. Second, the truck drivers who were responsible for the registration of movement records at the origin of movements might be dealers or unaware of the address of the destination. In these cases, the address as shown in the national identification card of the truck driver is usually used as the destination of the movement. A more consistent and reliable record of dairy cattle movements should be emphasized as it can be useful for disease investigation and outbreak controls in the region. Regarding this limitation, interpretation of the dairy cattle movement pattern, especially for the movements to destination premises rather than abattoir, should be cautiously made together with the movements for the purpose of growing and selling.

Social network analysis was conducted through quantification of metrics to investigate the hidden structures of subnetworks by stratification and to identify districts with an increased risk of infection and spread of disease. These network metrics provided a fast and easy way to identify districts that were highly active and influential to other districts in the network. For example, Mueang Lamphun in Lamphun province showed a high degree and high eigen centrality in several networks, which could be the hub of receiving and distributing male dairy calves for fattening and slaughtering in the region. In addition, Ban Thi in Lamphun province showed the highest betweenness, which could be associated with its geographical location as it is in proximity with several dairy cattle crowded areas in Lamphun and Chiang Mai provinces. Several large dairy farms with >100 milking cows are located in the identified districts in central and other regions of the country (14), which were considered influential hot spots of origins of dairy cattle movements to the northern region of Thailand. Other influential districts located in northern Thailand are areas with a high density of smallholder dairy farms containing 20–100 milking cows (8). These districts are considered either hot spots of destinations as they mainly received dairy cattle for growing and selling or hot spots of origins of dairy cattle movements within the northern region of Thailand as they also distributed dairy cattle for growing and selling in other districts within the region. Moreover, our networks are north-centric; therefore, the influential districts identified from regions outside of the northern region need complete movement data from all regions to reevaluate their connectivity among these networks. Our approach of utilizing network metrics to identify influential districts can be easily applied once such data become available.

We found that the movement for slaughtering and the movement for growing and selling created distinctive networks with important implications for disease control and contact tracing. The network analysis identified potential targets to direct control efforts in more than 100 districts. Stratifying networks based on the purpose of movement helped realize the hidden pattern masked by a large number of slaughtering movements. More importantly, their differences in the network characteristics suggested that, during an epidemic, the spread of disease may be faster for the dense and localized slaughtering network than the growing and selling network. On the contrary, it may be difficult to implement disease control and contact tracing in the growing and selling network due to the large number of nodes involved. Further investigation may include incorporating geographical information because cattle producers need to travel across different provinces to move animals from one location to another. Incorporating road traffic and traveling routes could significantly improve the estimation of network structures. In addition, epidemic data can be simulated on the observed movement network to benchmark the performance of network metrics on recovering the chain of disease transmissions.

Even though several findings are reported in the current study, interpretation of the results should be cautiously made due to several limitations. One limitation is that only data on legal movements of dairy cattle were included for the analysis in the current study. Movements of beef cattle, buffaloes, and other non-ruminant animals can potentially contribute to the disease transmission in dairy cattle because most infectious diseases are not specific for only dairy cattle. Therefore, the implication of results from the current study for the transmission of diseases such as FMD, which can be transmitted through the movement of different domestic animals or through other mechanical vectors, can be very limited. Moreover, only records of movements between districts were available for analysis. Movements of dairy cattle within districts might more frequently occur, which can significantly contribute to the disease transmission between villages within each district. Information bias on types of premises of origins and destinations, and the primary purpose of the movement could occur in the current study because the records of these pieces of information could be subjectively and inconsistently made by the truck drivers. This bias can be minimized by applying a criterion to be used for recording these data at the movement registration, which can consequently improve the validity and accuracy of the data analysis.

## Conclusion

Dairy cattle movements in the northern region of Thailand from 2010 to 2017 were analyzed. Decreasing trends in both dairy cattle movements and the number of cattle moved were observed from 2010 to 2016. In 2017, the movements of dairy cows for growing and selling increased from previous years. From the network analysis, several influential districts in northern and other regions were identified. These districts are key areas with potential for disease transmission among dairy cattle in the northern region of Thailand and the spreading of infectious diseases across regions in Thailand.

## Data availability statement

The data analyzed in this study is subject to the following licenses/restrictions: The raw data supporting the conclusions of this article will be made available by the authors, without undue reservation. Requests to access these datasets should be directed to wang1927@umn.edu.

## Author contributions

SB, SW, and KV designed the study. TS and AK collected, retrieved, and curated the data. YW analyzed the data. SB and YW wrote the manuscript. All authors read and approved the final manuscript.

## Funding

This work was funded by the Faculty of Veterinary Medicine, Chiang Mai University and the College of Veterinary Medicine, University of Minnesota (R000025322, 2020).

## Conflict of interest

The authors declare that the research was conducted in the absence of any commercial or financial relationships that could be construed as a potential conflict of interest.

## Publisher's note

All claims expressed in this article are solely those of the authors and do not necessarily represent those of their affiliated organizations, or those of the publisher, the editors and the reviewers. Any product that may be evaluated in this article, or claim that may be made by its manufacturer, is not guaranteed or endorsed by the publisher.
