# Supplementary figures and images for: Analysis of dairy cattle movements in the northern region of Thailand
Source: Front Vet Sci. 2022 Oct 4;9:961696. doi: 10.3389/fvets.2022.961696 (PMC9577029; doi:10.3389/fvets.2022.961696)

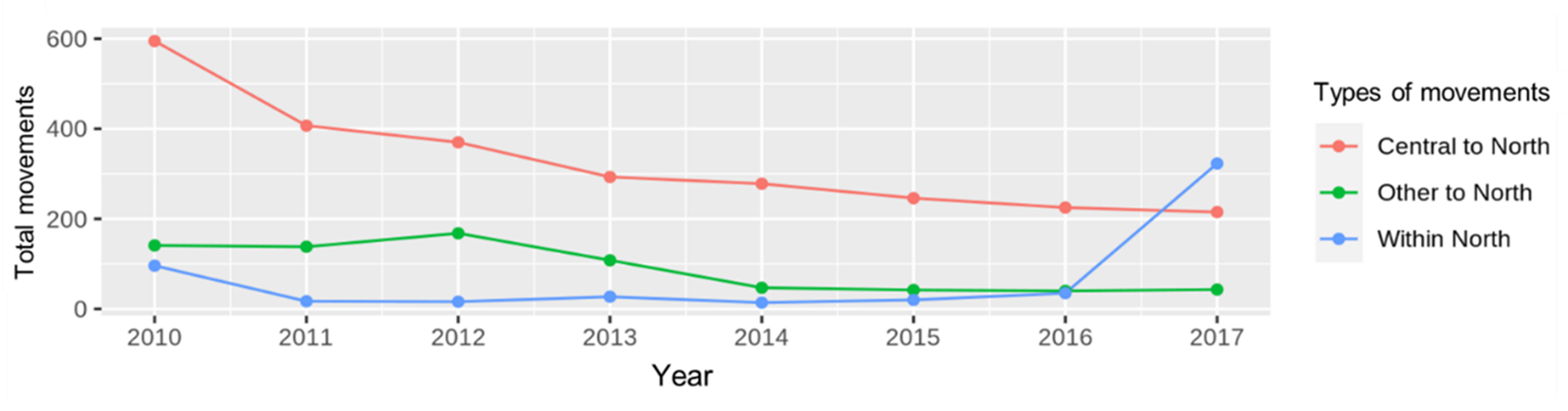

Supplement: Supplementary file 4 [file Image_1.tif]

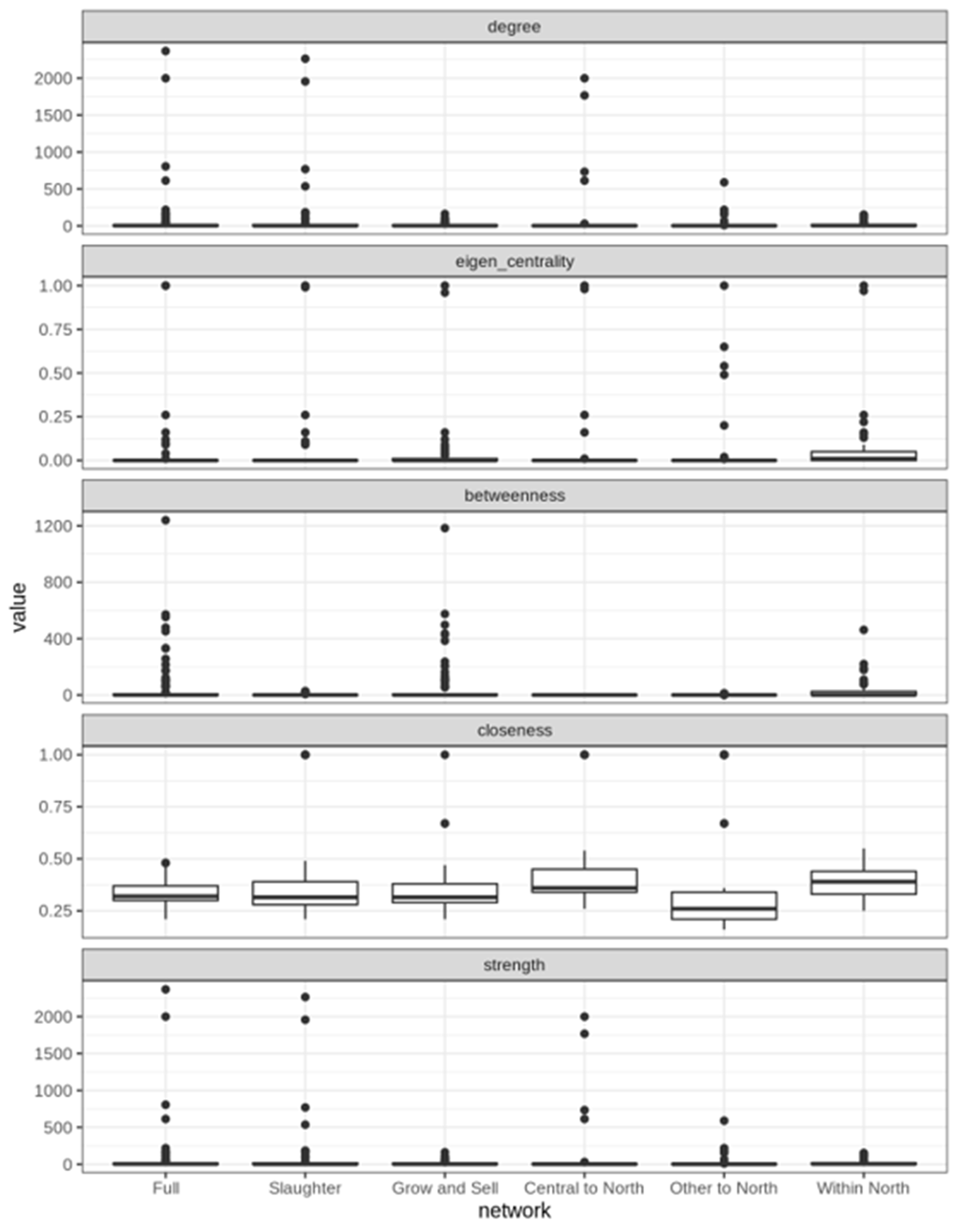

Supplement: Supplementary file 5 [file Image_2.tif]
